# Primary Orbital Reconstruction with Selective Laser Melting (SLM) of Patient-Specific Implants (PSIs): An Overview of 96 Surgically Treated Patients

**DOI:** 10.3390/jcm11123361

**Published:** 2022-06-11

**Authors:** Majeed Rana, Henriette L. Moellmann, Lara Schorn, Julian Lommen, Madiha Rana, Max Wilkat, Karsten Hufendiek

**Affiliations:** 1 Department of Oral and Maxillofacial Surgery, Heinrich Heine University Duesseldorf, Moorenstrasse 5, 40225 Duesseldorf, Germany; rana@med.uni-duesseldorf.de (M.R.); lara.schorn@med.uni-duesseldorf.de (L.S.); julian.lommen@med.uni-duesseldorf.de (J.L.); max.wilkat@med.uni-duesseldorf.de (M.W.); 2 Department of Psychology, University of Applied Sciences, Doberaner Weg 20, 22143 Hamburg, Germany; madiha.rana@euro-fh.de; 3 Department of Ophthalmology, Hannover Medical School, Carl-Neuberg-Strasse 1, 30625 Hannover, Germany; hufendiek.karsten@mh-hannover.de

**Keywords:** orbital reconstruction, selective laser melting, customized implant, 3D mesh, orbital wall fracture, intraoperative navigation

## Abstract

Contemporary advances in technology have allowed the transfer of knowledge from industrial laser melting systems to surgery; such an approach could increase the degree of accuracy in orbital restoration. The aim of this study was to examine the accuracy of selective laser melted PSIs (patient-specific implants) and navigation in primary orbital reconstruction. Ninety-six patients with orbital fractures were included in this study. Planned vs. achieved orbital volumes (a) and angles (b) were compared to the unaffected side (*n* = 96). The analysis included the overlay of post-treatment on planned images (iPlan 3.0.5, Brainlab^®^, Feldkirchen, Germany). The mean difference in orbital volume between the digitally planned orbit and the postoperative orbit was 29.16 cm^3^ (SD 3.54, presurgical) to 28.33 cm^3^ (SD 3.64, postsurgical, t = 5.00, df = 95.00; *p* < 0.001), resulting in a mean volume difference (planned vs. postop) of less than 1 cm^3^. A 3D analysis of the color mapping showed minor deviations compared to the mirrored unaffected side. The results suggested that primary reconstruction in complex orbital wall fractures can be routinely achieved with a high degree of accuracy by using selective laser melted orbital PSIs.

## 1. Introduction

Fractures of the facial skeleton are often the center of attention, due to their frequency and the complexity of the surgical reconstruction. The orbit is a susceptible region in the midface. Overall, up to 40% of craniomaxillofacial traumas are associated with orbital fractures [1,2]. The mode of action is variable, but orbital fractures may result from violent assaults, motor vehicle accidents or sports-related injuries [3,4,5]. External impact forces seem to cause a so-called ‘blowout’ [6]. Dependent on the type of impact—commonly following sports-related injuries—orbital floor fractures may be isolated injuries [7]. There is general agreement that these fractures should receive early treatment, usually within two weeks [6,8]. The clinical presentation following an orbital fracture is largely dependent on the extent and any other associated fractures of the facial skeleton. To treat or even prevent severe complications such as diplopia, hypoglobus or changes in facial geometry, a fracture reduction as close as possible to the original anatomy is mandatory [9,10]. The goals are to re-establish normal function, aesthetics and accomplish appropriate reconstruction of the midface [9]. The contemporary standard in many institutions is surgical restoration with individually bent or preformed meshes [11,12]. To avoid inadequate surgical treatment, a high-resolution preoperative CT scan and digital planning could be useful and could prevent post-procedure asymmetry [5,13,14]. To deal with these issues, patient-specific three-dimensional mesh fabrication and image-guided navigation are options to perform complex orbital rehabilitations [10]. Advances in these technologies have made it possible to achieve increasing degrees of accuracy in the treatment of orbital deformities. This tactic is associated with knowledge of specific anatomical circumstances, decreased operative times and precise control of implant position [15,16].

Preliminary results indicate that this technique has the potential to decrease the angle and orbital volume deviation from the unaffected to the distracted orbital space [17]. The focus of this single-center prospective analysis is to present our experience and highlight the potential advantages of orbital SLM PSIs (selective laser melted patient-specific implants) in the primary reconstruction of complex orbital fractures. This could help clinicians optimize the digital and clinical workflow for orbital SLM PSIs.

## 2. Materials and Methods

This study analyzes the results of unilateral orbital fractures treated at the Department of Craniomaxillofacial Surgery, Hannover Medical School, and the Department of Oral and Maxillofacial Surgery, Heinrich Heine University of Duesseldorf, Germany, between October 2013 and December 2017 using orbital PSIs. There was only one primary surgeon for all patients (author MR), and no other method of orbital reconstruction was used during that time.

**Figure 1:**
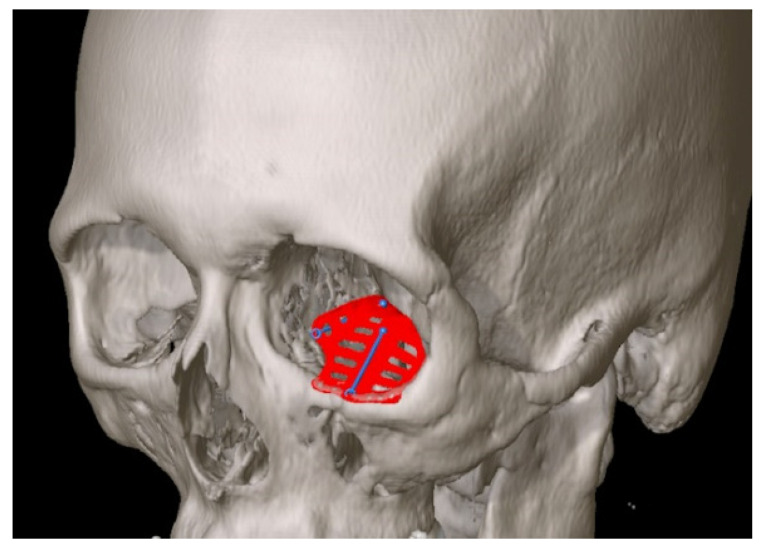
A selective laser melted patient-specific implant designed for the left orbit, two wall reconstruction. A horizontal drainage system is incorporated throughout. Navigational landmarks and guides are provided to facilitate implant placement with intraoperative navigation. Screw holes are placed anteriorly for fixation to the inferior orbital rim.

**Figure 2:**
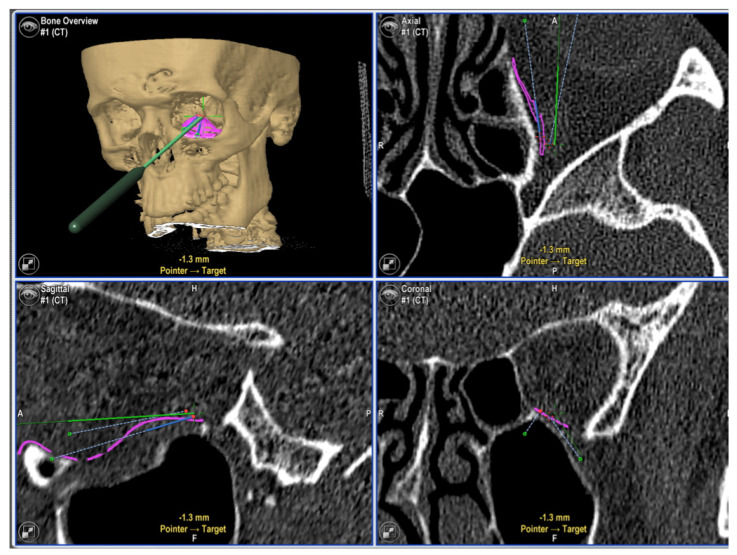
Intraoperative navigation used to confirm correct implant position. (**Upper left**): pointer resting on a navigational landmark of the PSI. (**Upper right**, **Lower right** and **left**): screenshots showing position of pointer in axial, sagittal and coronal views (tip of pointer is represented by the centre of the green crosshairs).

**Figure 3:**
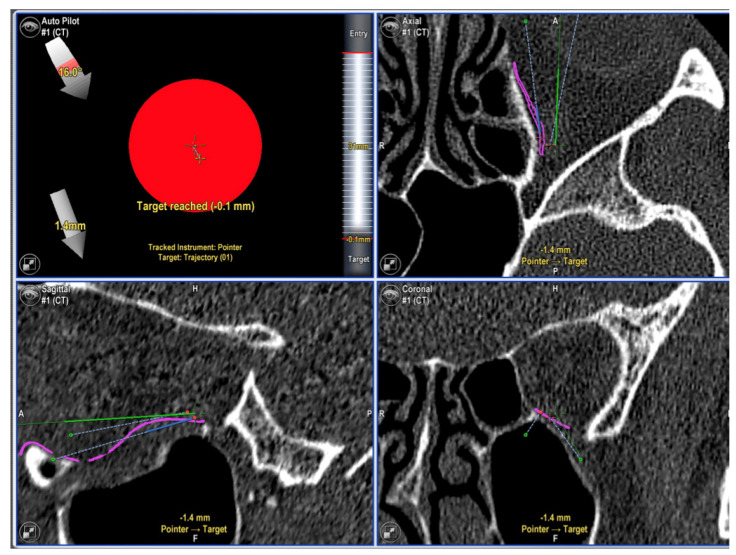
(**Upper left**): screenshot showing pointer has reached the navigation landmark. (**Upper right**, **Lower right** and **left**): screenshots showing position of pointer in axial, sagittal and coronal views (tip of pointer is represented by the centre of the green crosshairs).

Patients were included if they had reconstruction for primary unilateral orbital deformities, for either first stage surgery or second stage after treatment of zygomatic/midface fracture, using computer-assisted treatment (Figure 1) during the study period. In addition, the patients fulfilled the following inclusion criteria: (a) patients older than 18 years, (b) indication for orbital reconstruction true to origin planning (indications were given in case of double vision, enophthalmos, hypoglobus and defect size >10 mm), (c) intraoperative image-controlled reconstruction (Figure 2 and Figure 3), (d) existence of pre-surgery CT or CBCT, (e) patient letter of agreement, (f) adequate follow-up care and examination and (g) existing vision in the affected eye. In addition, the indications for using computer-assisted navigation, as used at the Hannover Medical School, Germany, had to be fulfilled. These indications included the following:

- Fractures of the medial orbital wall;
- Fractures of the posterior third of the orbital floor;
- Complex comminuted orbital fractures;
- Orbital wall fractures, including the transition zone between medial wall and orbital floor.

Exclusion criteria included secondary or tertiary reconstruction of the orbit, pretraumatic anophthalmic orbit or amaurosis and participants aged under 18 years.

The two outcome variables were the orbital volume and the intraorbital implant angulation. As a guiding aim, we planned the orbital restoration based on the unaffected side (in terms of size and shape). We looked at the details of the final implant position, and we quantified the orbital pre- and postoperative volume to validate accuracy. In addition, we measured the angles (anterior, medial and posterior) in the coronal view of the 3-dimensional imaging. Plate placement and volume measurement were evaluated using the atlas-based 3-dimensional software iPlan 3.0.5 (Brainlab^®^, Feldkirchen, Germany). The absolute mean difference as well as the standard deviation were calculated for the final statistical calculation.

In detail, the 3D evaluation was performed as follows: the raw data of all pre- and postoperative CT and CBCT scans (layer thickness <1 mm) were imported to iPlan CMF 3.0.5 (Brainlab, Feldkirchen, Germany). All data sets were aligned according to the Frankfurt Horizontal and Median Sagittal Plane.

The defect size of the orbital floor and the medial orbital wall was measured as the maximum diameter in the coronary and sagittal plane. The respective distances were expressed in millimeters. For (1) the healthy, unaffected site and (2) the fracture affected and reconstructed post-operative site, an angle measurement was carried out between the medial orbital wall and the orbital floor in the coronary layer using three defined landmarks: the cranial boundary of the medial wall, the transition zone between the medial wall and the orbital floor, and the transition zone between the orbital floor and the lateral wall. The measurements were carried out in the anterior, middle and posterior regions of the orbit. These were defined in the sagittal plane by (1) the anterior bony orbital margin, (2) the posterior bony boundary (the so-called posterior ledge) and (3) the exact half of the sagittal distance between these two.

For the volume measurement of (1) the healthy, unaffected site, (2) the fracture affected preoperative site, and (3) the fracture affected site post-operatively after reconstruction, the orbital cavities were segmented with the program iPlan CMF 3.0.5. Via the “object creation“ tool, automated segmentation, on the basis of anatomical models, was performed. This first step of segmentation was then manually verified by dragging the outlines of the segmented orbit via the smart shaper option to define the volumes individually. The volume of the created 3D object was then calculated and displayed by the software.

Additional study variables included the following (Table 1): gender, etiology of defect, type of fracture, number of injured orbital walls (single: one wall; multi-wall: more than one wall), indication for surgery, navigational tools used, and average defect size in coronal and sagittal view in mm. We made a note if there was a double operation procedure (e.g., first, positioning of the midfacial bony frame and, secondly, restoring the orbital with a PSI).

Preoperative conventional high-resolution computed tomography (CT) and/or Cone Beam computed tomography (CBCT) and its DICOM scan data were generated. For the implant creating procedure, we used iPlan^®^ CMF 3.0.5 (Brainlab^®^, Feldkirchen, Germany) and the program Geomagic Freeform^®^ Plus (Morrisville, NC, USA) as previously described (Figure 1). An accurate transfer of the virtual plan to a precise PSI is mandatory for success. Most of the planning processes were carried out by the surgeon. For very complex cases, we liaised closely with the engineers (KLS-Martin^®^, Tuttlingen, Germany), through web meetings or telephone calls. After planning, the production process itself took up to a maximum of 5 days. All PSIs were manufactured in a selective laser melting procedure using titanium alloy Ti6Al4V in an argon atmosphere using a Concept Laser M2 (GE, Boston, MA, USA).

At the time of surgery, all patients were approached via a retroseptal, transconjunctival incision without a lateral canthotomy. During the procedure, intraoperative navigation (Kick, Brainlab^®^, Feldkirchen, Germany) was in use to assess the correct implant position within less than 1 mm of the targeted reconstruction area (Figure 2 and Figure 3). Proper positioning of the bony segments and internal orbit were confirmed with the following protocol: infraorbital rim, lateral rim, orbital floor, medial internal orbit/postero-medial orbital bulge, lateral internal orbit, posterior orbit and globe projection. The previously manufactured and inserted PSI was locked after position control with one or two 1.3 mm titanium micro screws (DePuy Synthes, Oberdorf, Switzerland).

All patients received a postoperative Cone beam scan (NewTom DVT 9000, Deutschland AG, Marburg, Germany) or a CT scan. The postoperative images were superimposed onto the preoperative images and were analyzed to assess if the reconstituted position was equal to the planned position. Differences in the orbital contour, the PSI position and the angular deviations were noted.

The data were analyzed with IBM SPSS Statistics for Macintosh, Version 28.0.1.1 (IBM Corp., Armonk, NY, USA). Each study variable was computed using descriptive statistics. For testing the differences between the planned vs. achieved orbital volume and the three angles (anterior, medial, and posterior), a matched pairs t-test was used to assess the differences. An α-level of 0.05 was set as the level of statistical significance. All *p*-values were two sided.

## 3. Results

One hundred patients with complex orbital, unilateral primary post-traumatic bone fractures received SLM implants with intraoperative navigation. Ninety-six patients fulfilled all the inclusion criteria by having the complete therapy data available, including follow-up post treatment up to one year. An overview about patient demographics, injury causes and measurements is demonstrated in Table 1. The study cohort (included patients) was composed of 62 males and 34 females. The average age was 50.25 years.

Diagnosis was validated by imaging (CT/ CBCT). A total of 71.9% of all included patients had an isolated orbital fracture, while all the others had a combined midface fracture (zygomaticomaxillary complex, naso-orbital-ethmoidal or panfacial fracture). In total, 20 out of 96 patients (20.8%) had a simple (one wall) fracture, while all the others had complex (more than one wall) fractures.

Concerning orbital fractures, the average defect sizes (measurement was performed at the largest fracture diameter in coronal and sagittal view) were 23.96 mm (SD 6.52) and 25.91 mm (SD 4.49) (Table 1).

**Figure 4:**
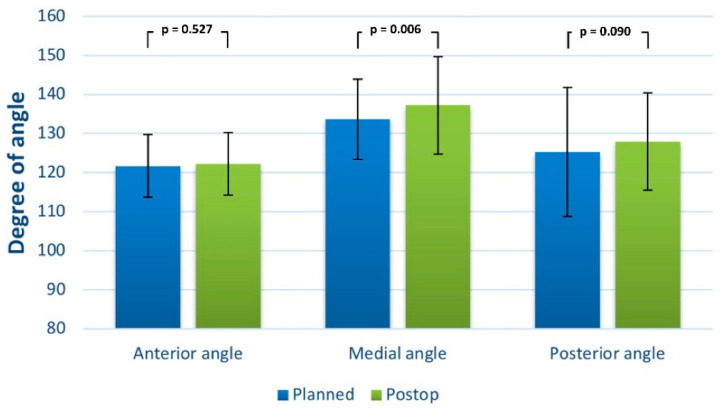
Bar graph showing the postoperative angular deviation from the unaffected orbit (planned reconstruction).

The orbital volume of the unaffected side ranged from 30.51 mL ± 2.94 mL in males to 26.70 mL ± 3.22 mL in females versus the volume of the affected side preoperatively from 30.08 mL ± 3.25 mL in males to 26.10 mL ± 2.84 mL in females (CT/CBCT). The mean difference in orbital volume between the digitally planned orbit and the postoperative orbit was 29.16 cm^3^ (SD 3.54; presurgical) to 28.33 cm3 (SD 3.64; postsurgical; *t* = 5.00; df = 95.00; *p* < 0.001), resulting in a mean volume difference (planned vs. postop) of less than 1 cm^3^. The mean difference between the planned and reached implant angulation (in coronal view) was 121.71° (SD 8.04) to 122.25° (SD 8.03) for the anterior angle (*t* = −0.635; df = 95.00; *p* = 0.527), 133.66° (SD 10.22) to 137.22° (SD 12.43) for the medial angle (*t* = −2.82; df = 95.00; *p* = 0.006) and 125.23° (SD 16.52) to 127.95° (SD 12.48) for the posterior angle (t = −1.71; df = 95.00; *p* = 0.090) (Figure 4).

The reconstructed orbital volume ranged from 29.70 ± 3.26 mL in males to 25.85 ± 2.95 mL in females (CBCT). A 3D analysis of the color mapping showed minor deviations compared to the mirrored unaffected side.

Table 2 compares operation times between different extended fractures and gives an overview about the median procedure time including navigation. It should be noted that all procedures were performed by a single operator; thus, the median procedure time should only measure the baseline and represents no valid statistical significance.

## 4. Discussion

Desirable long-term clinical outcomes could be achieved with the use of the correct radiographic modality and by restoring the exact orbital contoured volume [18,19,20]. This work showed the importance of ‘true-to-origin’ primary orbital reconstruction with PSIs. Good cosmetic and functional results can be achieved with early repair [21]. Digital planning and computer-assisted surgery are particularly helpful in large and complex facial deformities [16,22,23]. Particularly concerning accuracy during orbital reconstruction, computer-assisted surgery with preoperative planning helps to guide surgeons during surgery [24]. Furthermore, pre-bent implants using 3D-printed orbital anatomical models have been shown to provide a more accurate reconstruction of the orbital floor and a better functional outcome than a standardized, intraoperatively adapted titanium implant [25].

In our study, comprising a patient collective of 96 patients undergoing primary orbital reconstruction due to a traumatized orbit, we used CAD/CAM patient-specific implants. Navigational guides and rulers could be built into the implant. These navigational target points enable much better spatial orientation and feedback on whether the implant is actually where it is supposed to be [10]. As the pointer traverses along the trajectory guides, the navigation system can confirm that certain points are in the correct position and also that the trajectory is correct. These advantages lead to an exceedingly accurate implant position that can be placed without additional intraoperative CT scans, so there is no additional intraoperative radiation. The combination of up-to-date techniques offers the best possible results, even in complex cases [26]. However, the abovementioned techniques of virtual planning and intraoperative navigation require certain expert knowledge. Thereby, our study is limited due to one single planning and operating surgeon, which makes the transfer of the results especially difficult in situations using less experienced, younger surgeons. Moreover, it has been shown that the learning curve of virtual planning for orbital fractures is steep [27], and intraoperative navigation offers more security for experienced as well as inexperienced surgeons [28].

There are multiple goals of treatment for complex orbital deformities, which include avoiding complications such as visual disturbances, compromised facial aesthetics, extraocular muscle restriction and enophthalmos. Such complications can prolong the recovery journey and can affect the health-related quality of life. In very large defects, the posterior ledge often generates adequate footing in the deep orbit, which can facilitate the appropriate placement of the implant. Reaching this poorly visualized anatomic area can be very challenging, and intraoperative navigation can lead to success [29]. In addition, due to the so-called trajectory guides and rulers, the use of SLM PSIs could prevent possible adverse insertion effects on soft or hard tissues because of sharp edges or displaced mesh [17]. In addition, the structure of the surfaces of PSIs and, thus, their susceptibility to biofilm formation might play a role in their tissue integration and possible adverse effects [30,31], which gives the need for further research.

Our long-term results are consistent with other centers and show no disadvantage when compared to other surgical procedures [6,32]. We believe that possible long-term complications, such as diplopia, hypoglobus, enophthalmos, facial disproportion and decreased globe motility, could not always be prevented by any medical procedure known today; surgeons have no influence on fat positioning, muscle or connective tissue atrophy. However, the contemporary clinical work up has the potential to rebuild, as best as possible, the pre-accidental orbital bone position. In our study, we used the gold standard of mirroring the healthy unaffected site to define the pre-accidental orbital bone position. However, there might be other options to define the aim for perfect orbital reconstruction, such as the statistical shape model [33]. Nonetheless, in our study, we could show that the digitally planned reconstruction result can be achieved in reality with a high degree of accuracy, thanks to patient-specific implant design using SLM in combination with intraoperative real-time navigation.

## 5. Conclusions

This prospective study shows that complex orbital fractures can be reconstructed with a high degree of accuracy concerning the planned and postoperative implant fit. The digital workflow and computer-assisted surgery (analysis, preoperative planning and production, as well as intraoperative navigation) can provide a standard procedure. After a few years of clinical use, we believe that this technique is now suitable for daily use by clinical teams in trauma centers. However, the costs of the implant as well as the navigation system costs may preclude its widespread use in the near future and the entire world.

## Figures and Tables

**Table 1:** Study variables for included patients (*n* = 96).

Variables	Number of Patients
**Self-reported sex**	
Female	34
Male	62
**Etiology of defects**	
Traffic accident	14
Assault or Violence	22
Horse-associated accident	7
Golf ball hit	1
Bike spill	18
Stumble spill	18
Other cause	16
**Type of traumatic injury**	
Isolated orbital fracture	69
Zygomaticomaxillary complex, naso-orbital-ethmoidal, panfacial	27
**Number of stages for surgery**	
One stage procedure	47
Two stage procedure	49
**Wall types for reconstruction**	
Single wall	20
Two wall	76
Indication for surgery *	
Double vision initially	15
Enophthalmos	53
Hypoglobus	7
Exopthalmos	13
Hypaesthesia	1
Defect size and degree of dislocation	63
**Surgical access**	
Transconjunctival, retroseptal	All (96)
**Navigation tools**	
Calvarian screws	8
Dental splint	88
**Average defect size in mm**	**mean (SD)**
Coronal	23.96 (6.52)
Sagittal	25.91 (4.49)

* Note: the same patient can contribute to more than one category.

**Table 2:** Median procedure time including navigation in minutes (note: data are based on only one single operator to give a baseline).

	Mean	SD	Min	Max
One-wall fracture	110	61.20	42	270
Multi-wall fracture	118	83.77	47	480
Combination of panfacial and orbital restoration	164	139.86	50	600

## Data Availability

The data presented in this study are available on request from the corresponding author. The data are not publicly available due to privacy regulations.
